# Recent Advances in the Study of Bipolar/Rod-Shaped Microglia and their Roles in Neurodegeneration

**DOI:** 10.3389/fnagi.2017.00128

**Published:** 2017-05-04

**Authors:** Ngan Pan Bennett Au, Chi Him Eddie Ma

**Affiliations:** ^1^Department of Biomedical Sciences, City University of Hong KongKowloon Tong, Hong Kong; ^2^Centre for Biosystems, Neuroscience, and Nanotechnology, City University of Hong KongKowloon Tong, Hong Kong; ^3^State Key Laboratory in Marine Pollution, City University of Hong KongKowloon Tong, Hong Kong

**Keywords:** bipolar/rod-shaped microglia, amoeboid microglia, ramified microglia, neurodegenerative diseases, synapse

## Abstract

Microglia are the resident immune cells of the central nervous system (CNS) and they contribute to primary inflammatory responses following CNS injuries. The morphology of microglia is closely associated with their functional activities. Most previous research efforts have attempted to delineate the role of ramified and amoeboid microglia in the pathogenesis of neurodegenerative diseases. In addition to ramified and amoeboid microglia, bipolar/rod-shaped microglia were first described by Franz Nissl in 1899 and their presence in the brain was closely associated with the pathology of infectious diseases and sleeping disorders. However, studies relating to bipolar/rod-shaped microglia are very limited, largely due to the lack of appropriate *in vitro* and *in vivo* experimental models. Recent studies have reported the formation of bipolar/rod-shaped microglia trains in *in vivo* models of CNS injury, including diffuse brain injury, focal transient ischemia, optic nerve transection and laser-induced ocular hypertension (OHT). These bipolar/rod-shaped microglia formed end-to-end alignments in close proximity to the adjacent injured axons, but they showed no interactions with blood vessels or other types of glial cell. Recent studies have also reported on a highly reproducible *in vitro* culture model system to enrich bipolar/rod-shaped microglia that acts as a powerful tool with which to characterize this form of microglia. The molecular aspects of bipolar/rod-shaped microglia are of great interest in the field of CNS repair. This review article focuses on studies relating to the morphology and transformation of microglia into the bipolar/rod-shaped form, along with the differential gene expression and spatial distribution of bipolar/rod-shaped microglia in normal and pathological CNSs. The spatial arrangement of bipolar/rod-shaped microglia is crucial in the reorganization and remodeling of neuronal and synaptic circuitry following CNS injuries. Finally, we discuss the potential neuroprotective roles of bipolar/rod-shaped microglia, and the possibility of transforming ramified/amoeboid microglia into bipolar/rod-shaped microglia. This will be of considerable clinical benefit in the development of novel therapeutic strategies for treating various neurodegenerative diseases and promoting CNS repair after injury.

## Introduction

Microglia are generally considered as phenotypically diverse immune cells which reside in the central nervous system (CNS). The morphological changes of microglia are closely associated with their function and the microenvironment in which they reside (Chamak and Mallat, 1991; Suzumura et al., 1991; Szabo and Gulya, 2013; Tam and Ma, 2014; Tam et al., 2016). Under normal physiological conditions, microglia adopt a “surveying” phenotype, referred to as “ramified microglia” with compact cell bodies and elongated branching processes (Hanisch and Kettenmann, 2007). Ramified microglia frequently extend and retract their highly motile processes so that they can actively sense and survey their microenvironment to detect subtle changes, but without disrupting the existing neuronal circuitry (Nimmerjahn et al., 2005). Ramified microglia in the immediate vicinity of micro-lesions respond rapidly and quickly transform into an active state, thus allowing them to migrate towards the site of injury (Davalos et al., 2005; Nimmerjahn et al., 2005). Ramified microglia first thicken, withdraw their processes, enlarge their cell bodies, and subsequently transform into an amoeboid morphology. Amoeboid microglia are regarded as “activated” microglia since they are responsible for antigen presentation, the production of an exhaustive list of inflammatory cytokines, chemokines and neurotrophic factors and are responsible for the removal of cellular debris by phagocytosis (Wyss-Coray and Mucke, 2002; Glass et al., 2010). Recent studies have also shown that amoeboid microglia are closely associated with neurological disorders such as brain injuries, Alzheimer’s disease (AD), Parkinson’s disease (PD) and Huntington’s disease (HD) in which chronic neuroinflammation is usually observed (Glass et al., 2010; Krause and Müller, 2010; Smith et al., 2012). Amoeboid microglia are also detected in early and late stages of the onset of neurodegenerative diseases (Lynch, 2009). Studying the association of microglia with disease progression is therefore very attractive to neuroscientists, particularly in terms of neurodegeneration and neuroprotection.

Over the past few decades, most studies have focused upon the well-characterized ramified and amoeboid microglia, and studies of bipolar/rod-shaped microglia remain scarce due to the lack of a well-established *in vitro* culture system and a highly reproducible *in vivo* animal model with which to study this form of microglia. There is compelling evidence to suggest that bipolar/rod-shaped microglia play a pivotal role in CNS repair since they align end-to-end along with the damaged axons following traumatic brain injury (Ziebell et al., 2012; Taylor et al., 2014). Bipolar/rod-shaped microglia are also highly phagocytic and proliferative in nature (Ziebell et al., 2012; Tam and Ma, 2014), and are involved in the internalization of degenerating neurons following CNS injury (Yuan et al., 2015). Bipolar/rod-shaped microglia express a distinctive transcriptional profile in response to immunological stimuli such as lipopolysaccharides (LPS), and quickly transform into amoeboid microglia (Tam and Ma, 2014; Tam et al., 2016). This suggests that bipolar/rod-shaped microglia might be a transitional stage between the ramified and amoeboid microglia (Suzumura et al., 1990; Bohatschek et al., 2001; Jonas et al., 2012). A recent case study involving autopsy of over 160 patients showed that the presence of bipolar/rod-shaped microglia in the hippocampus and cerebral cortex was directly related to an AD cohort with aging and AD-related pathology such as dementia, and the formation of senile plaques and neurofibrillary tangles (NFTs; Bachstetter et al., 2015, 2017).

In this review article, we summarize our current understanding of bipolar/rod-shaped microglia based upon recent studies which have defined the role of this form of microglia in neurological diseases. We also discuss the potential neuroprotective role of bipolar/rod-shaped microglia in neurodegenerative diseases. Accumulating evidence suggests that the formation of bipolar/rod-shaped microglia might be potentially beneficial, not only in protecting the CNS neurons against progressive neuronal degeneration induced by chronic neuroinflammation, but also in helping with the reorganization of neuronal circuitry. This review aims to provide a more in-depth understanding of bipolar/rod-shaped microglia in order to provide new focus in the development of therapeutic strategies for neurodegenerative diseases.

## The Discovery and Identification of Bipolar/Rod-Shaped Microglia

Bipolar/rod-shaped microglia were first identified as an activated form of microglia by Nissl (1899) more than a century ago. In this original study, Nissl examined glia cell morphology in cerebral cortices from patients who suffered from paralytic dementia, and he described this form of microglia as “strung-out, extremely slim with infinitely long processes”. These bipolar/rod-shaped microglia extend their processes into pyramidal neuronal layers and are aligned with the growing tips of dendrites from nearby neurons (Nissl, 1899). Further studies showed that bipolar/rod-shaped microglia were also present in cerebral cortices in neurological disorders associated with typhus infections and syphilis, as well as sleeping disorders (Spielmeyer, 1922). The incidence of these neurological disorders was dramatically reduced, largely due to the discovery of penicillin. Thus, studies on this microglia subtype gradually reduced and researchers became unfamiliar with this form of microglia due to their rare occurrence in pathological brains (Graeber, 2010). This might account for our limited knowledge of bipolar/rod-shaped microglia, even though they were discovered more than a century ago.

Over the past two decades, bipolar/rod-shaped microglia were observed in several chronic neuropathological disorders, including viral encephalitis, lead encephalopathy and subacute sclerosing panencephalitis (SSPE). Patients with viral encephalitis exhibit microglial nodule formation in the brain which represents a hallmark pathological feature, regardless of the type and origin of the virus. This microglial nodule consists of a bundle of activated bipolar/rod-shaped microglia, reactive astrocytes, infiltrated macrophage and degenerating axons (Nelson et al., 2002). In a rat model of chronic lead (a heavy metal) intoxication, microglia were transformed into bipolar/rod-shaped with enlarged endoplasmic reticuli and a large quantity of cytoplasmic lipid inclusions after 9 months of continuous exposure to lead (Markov and Dimova, 1974). Autopsy of brains from SSPE patients further revealed that bipolar/rod-shaped microglia were the predominant form of microglia in the cortical area, as well as in the white matter (Wierzba-Bobrowicz et al., 2002; Boche et al., 2013). These studies collectively suggest that bipolar/rod-shaped microglia are often observed in various neuropathological disorders.

## Morphological Dynamics of Ramified, Bipolar/Rod-Shaped and Amoeboid Microglia

Bipolar/rod-shaped microglia are seldom observed in cultures owing to the small number of this microglia subtype in the overall microglia population (Suzumura et al., 1990; Bohatschek et al., 2001; Hoffmann et al., 2003; Szabo and Gulya, 2013). Primary microglia usually adopt an amoeboid morphology when cultured on non-coated surface (Suzumura et al., 1990; Kann et al., 2004). After treating with granulocyte-macrophage colony stimulating factor (GM-CSF), amoeboid microglia transformed into bipolar/rod-shaped microglia with elongated cell bodies and highly polarized processes following 5 days of incubation. The bipolar/rod-shaped microglia were highly proliferative compared to the amoeboid microglia (Suzumura et al., 1990).

Further study showed that the majority of microglia adopted ramified morphology when co-culturing on an astrocyte monolayer. The ramified microglia quickly transformed into bipolar/rod-shaped microglia within 3 h following the addition of rat brain lysate to the co-cultures, and these bipolar/rod-shaped microglia were gradually transformed into amoeboid microglia after 12 h of incubation. Interestingly, removal of brain lysate by washing off the culture medium induced a reverse transformation of amoeboid into bipolar/rod-shaped microglia within 24 h, and gradually transformed back into ramified microglia following 96 h of incubation without the brain lysate (Bohatschek et al., 2001).

One study showed that in rat hippocampal slice cultures, microglia adopted mixed and diverse morphological forms ranging from ramified, bipolar/rod-shaped to amoeboid 72 h following LPS challenge (Papageorgiou et al., 2016). Unlike other studies, the number of amoeboid microglia in hippocampal slice cultures remained unchanged after LPS stimulation (Gao et al., 2002; Qin L. et al., 2007). Also, the levels of pro-inflammatory cytokines such as interleukin 1 beta (IL-1β) and tumor necrosis factor α (TNFα) were significantly elevated, and the neuronal architecture remained largely intact without any detectable neuronal loss. In contrast, the number of amoeboid microglia was markedly increased when exposed to both LPS and interferon gamma (IFNγ), which demonstrated a significant neuronal loss (Papageorgiou et al., 2016). Transformation of bipolar/rod-shaped microglia into amoeboid microglia, and the induced neurotoxicity might involve the crosstalk between microglia and infiltrated leukocytes since IFNγ is mainly secreted by leukocytes but not by microglia (Papageorgiou et al., 2016).

Accumulating evidence suggests that morphological transformation of microglia involves the change in electrophysiological properties and intracellular calcium concentration ([Ca^2+^]i) of the microglial cells. Astrocytes are known to produce macrophage colony stimulating factor (M-CSF), which induces the transformation of amoeboid microglia into a mix of ramified (vast majority) and bipolar/rod-shaped microglia (small number) within few hours along with voltage-gated sodium (Na^+^) and outward potassium (K^+^) current increase in the transformed microglia (Frei et al., 1992; Korotzer and Cotman, 1992; Liu et al., 1994; Sievers et al., 1994; Eder et al., 1998; Kann et al., 2004). Ramified and bipolar/rod-shaped microglia returned to amoeboid morphology after washing out the astrocyte-conditioned medium (ACM; Eder et al., 1998). Further experiments showed the involvement of chloride (Cl^−^) channel in ramification of microglia. Cl^−^ channel blockers added to the ACM inhibited the transformation of microglia; however, blocking Na^+^ and K^+^ channels did not affect the ramification process (Eder et al., 1998). Ramification of microglia involves in membrane stretching (i.e., extension of processes) and cytoskeletal reorganization that might activate Cl^−^ channel. Stretching of microglial cell membrane under experimental condition was induced by a fine movement of recording patch pipette resulting in a current activation mediated mainly by Cl^−^ ions (Eder et al., 1998).

The change in [Ca^2+^]i serves as a signal transduction pathway to control various cellular events such as cell migration, proliferation, release of cytokines and morphological transformation of microglia (Möller, 2002; Farber and Kettenmann, 2006). Microglia express purinergic receptors and G protein-coupled receptor CD88 to regulate the [Ca^2+^]i of microglia (Farber and Kettenmann, 2006). Microglia irrespective of their morphology, including ramified and bipolar/rod-shaped microglia in ACM-treated microglia cultures, or amoeboid microglia on non-coated surface, showed a transient increase in [Ca^2+^]i in response to the agonists of purinergic receptor (i.e., ATP and UTP) and CD88 (i.e., Complement factor 5a, C5a), respectively (Möller et al., 2000; Kann et al., 2004). In contrast, LPS-induced activation of microglia resulted in a chronic increase in the basal [Ca^2+^]i, and suppression of UTP- and C5a-evoked transient increase in [Ca^2+^]i (Hoffmann et al., 2003). The UTP- and C5a-evoked transient increase in [Ca^2+^]i was successfully restored with the suppression of LPS-induced elevation of [Ca^2+^]i by BAPTA and AG126 (Hoffmann et al., 2003; Kann et al., 2004). The LPS-induced production of pro-inflammatory cytokines TNFα and IL-6, and nitric oxide (NO) by activated microglia requires the elevation of [Ca^2+^]i. The release of cytokine and NO were significantly reduced by the blockade of intracellular calcium release in LPS-treated microglia using BAPTA (Hoffmann et al., 2003). ACM-induced ramified and bipolar/rod-shaped microglia did not differ functionally from amoeboid microglia grown on non-coated surface. However, the LPS-stimulated microglia behave differently in regulating [Ca^2+^] (Möller et al., 2000; Hoffmann et al., 2003; Kann et al., 2004).

## Cell Culture Model Systems for Studying Bipolar/Rod-Shaped Microglia

The study of bipolar/rod-shaped microglia has been limited by the lack of *in vitro* and *in vivo* model systems with which to study this form of microglia. Unlike the case for bipolar/rod-shaped microglia, there are many well-defined cell culture systems available to purify ramified and amoeboid microglia, which provide us with a better understanding of the molecular and cellular properties of these microglia subtypes. In general, primary microglia cultured on a fibronectin-coated surface enrich ramified microglia, while microglia cultured on a laminin-coated surface enrich amoeboid microglia (Chamak and Mallat, 1991). Many studies have already taken advantage of this relatively simple culture system to examine how microglia respond to various factors such as ATP (Hide et al., 2000; Honda et al., 2001), LPS (Nakamura et al., 1999; Qin H. et al., 2007; Pannell et al., 2014), and cytokines (Merrill, 1991). Moreover, cell culture systems allow researchers to examine gene expression profiling (Duke et al., 2004; Horvath et al., 2008), along with cellular properties such as cell migration (Honda et al., 2001; Haynes et al., 2006), phagocytic activity (Townsend et al., 2005; Koizumi et al., 2007), electrophysiological properties (Eder et al., 1998) and intracellular calcium activity (Hoffmann et al., 2003; Kann et al., 2004) in different forms of microglia. In few studies, in which ramified and partially bipolar/rod-shaped microglia were induced within the same culture *in vitro*, the functional differences of these two distinct microglia phenotypes were not explored in detail (Eder et al., 1998; Möller et al., 2000; Kann et al., 2004).

**Figure 1:**
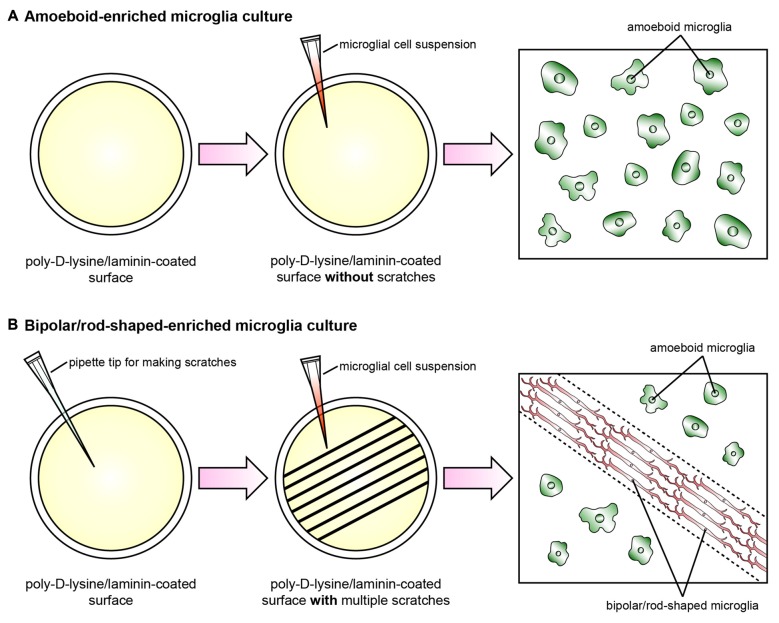
**Schematic diagram illustrating the cell culture model systems to enrich amoeboid and bipolar/rod-shaped microglia.** Cerebral cortices dissected from postnatal mice were trypsinized and dissociated mechanically, and the cell suspension was plated onto a poly-D-lysine-coated culture flask. The microglia were then isolated from the mixed primary cortical cultures after 10–12 days by gently shaking. The purity of the microglia cultures prepared by this method was >99% (Tam and Ma, 2014; Tam et al., 2016). **(A)** The vast majority of primary microglia maintained an amoeboid-like morphology after culturing on a poly-D-lysine/laminin-coated surface without any physical scratches. **(B)** Multiple physical scratches generated by a P200 pipette tip removed the laminin coating, which created laminin-free zones between the non-scratched areas. The primary microglia actively migrated to the scratched area and formed trains of bipolar/rod-shaped microglia (red) in parallel with the direction of the scratches (dotted lines). By contrast, the microglia colonized in the non-scratched surfaces maintained amoeboid morphology (green) while the laminin coating was still intact.

Recently, we established a cost-effective and highly reproducible method with which to enrich bipolar/rod-shaped microglia *in vitro* (Tam and Ma, 2014; Tam et al., 2016) and thus study gene expression profiles and characterize morphological changes systemically. Primary microglia were isolated from postnatal mice and grown on a poly-D-lysine and laminin-coated surface with multiple scratches (Figure 1). Microglia actively migrated towards the scratched surface (Tam and Ma, 2014) where the laminin coating was scratched off (Tam et al., 2016) during the initial hours after cell seeding, and microglia colonized upon the laminin-free scratched surface developed elongated and bipolar processes, beginning from the first day *in vitro* (DIV; Tam and Ma, 2014; Tam et al., 2016). Bipolar/rod-shaped microglia with elongated processes quickly formed end-to-end alignments on the scratched poly-D-lysine and laminin-coated surface after 1 DIV (Tam and Ma, 2014). In contrast, although trains of bipolar/rod-shaped microglia could be formed on scratched surface pre-coated with poly-D-lysine only, these trains of bipolar/rod-shaped microglia failed to maintain and the microglia alignments disappeared after 3 DIV. This suggests that laminin is crucial in stabilizing the formation of microglia alignments since the trains of bipolar/rod-shaped microglia could be maintained for at least 6 DIV on the scratched poly-D-lysine and laminin-coated surface (Tam et al., 2016). In rats, bipolar/rod-shaped microglia aligned end-to-end at the injury site within 1 day of diffuse brain injury *in vivo* (Ziebell et al., 2012). In line with an *in vivo* study on human pathological brains (Wierzba-Bobrowicz et al., 2002), bipolar/rod-shaped microglia in culture showed high immunoreactivity to proliferating cell nuclear antigen (PCNA, a cell proliferation marker), indicating that they were highly proliferative. The gene expression of both M1 (i.e., *Tnf, Il-1b, Cd32* and *Cd86*) and M2 (i.e., *Il-10* and *Tgf-β*) markers was markedly reduced in bipolar/rod-shaped microglia at the time when trains of microglia were formed (i.e., 2 DIV). In contrast, amoeboid microglia are known to produce significantly higher levels of pro-inflammatory cytokines compared to bipolar/rod-shaped microglia (Tam and Ma, 2014). Amoeboid microglia colonized in the non-scratched area (i.e., laminin-coated surface), thus demonstrating their ability to digest the laminin coating (Stolzing et al., 2002; Tam et al., 2016) with the up-regulation of laminin-cleaving proteins, Adam9 and Ctss, compared to bipolar/rod-shaped microglia which colonized in the laminin-free scratched surface (Tam et al., 2016). In addition, bipolar/rod-shaped microglia were morphologically dynamic and could rapidly transform into amoeboid microglia within 30 min in response to LPS (i.e., a M1 stimulus). This involved the up-regulation of pro-inflammatory cytokines (*Il-1b* and *Tnf*) and activation of the Jak/Stat3 signaling pathway (Tam and Ma, 2014). Amoeboid microglia which had transformed from bipolar/rod-shaped microglia regained the ability to cleave laminin via the up-regulation of the laminin-cleaving proteins, Adam9 and Ctss, while their gene expression in LPS-treated amoeboid microglia-enriched cultures remained unchanged (Tam et al., 2016). Taken together, our recently established cell culture system successfully enriched bipolar/rod-shaped microglia forming end-to-end alignments *in vitro* (Tam and Ma, 2014; Tam et al., 2016) that resemble the spatial arrangement of trains of bipolar/rod-shaped microglia after CNS injury *in vivo* (Ziebell et al., 2012; Taylor et al., 2014; Yuan et al., 2015). However, bipolar/rod-shaped microglia formed after GM-CSF and brain lysate stimulation did not form end-to-end alignments (Suzumura et al., 1990; Bohatschek et al., 2001), which indicate that a physical scratched surface (perhaps mimicking the site of injury) is required for the end-to-end alignment formation.

## Bipolar/Rod-Shaped Microglia in Brain Injury

Colonization and trains of bipolar/rod-shaped microglia are not normally observed in the naïve rat brain (Taylor et al., 2014), and are only occasionally found in the cerebral cortex and thalamus of rodent brain (Lawson et al., 1990; Taylor et al., 2014). Following midline fluid percussion injury (mFPI), an animal model of traumatic brain injury, ramified microglia which used to be evenly distributed throughout the cortical area were activated and adopted a “rod-like” morphology. The cell bodies of these “rod-like” microglia were elongated and their primary processes projected toward the two ends, and formed trains of bipolar/rod-shaped microglia (Taylor et al., 2014). These bipolar/rod-shaped microglia appeared to be highly polarized in response to brain injury. In rats, the vast majority of bipolar/rod-shaped microglia started colonizing the primary somatosensory barrel field (S1BF) region in the cerebral cortex to form end-to-end alignments perpendicular to the dural surface across the cortical layers as early as 1-day post injury (dpi). An increased number of bipolar/rod-shaped microglia, with significant microglia alignments, were observed at 2 dpi, which became more prominent by 7 dpi (Ziebell et al., 2012). More importantly, these bipolar/rod-shaped microglia were phenotypically distinct from other forms of microglia (i.e., ramified and amoeboid microglia) with significant increase in cell length to width ratio, and reduced number and length of side branches (Taylor et al., 2014). Most of the bipolar/rod-shaped microglia exhibited high immunoreactivity to ED1 (a phagocytic marker), but only a few showed immunoreactivity to OX6 (a major histocompatibility complex (MHC) class II marker; Ziebell et al., 2012). This indicated that bipolar/rod-shaped microglia are highly phagocytic (Bauer et al., 1994; Cho et al., 2006). Interestingly, bipolar/rod-shaped microglia with high phagocytic activity were not involved in the clearance of myelin debris since no internalized myelin was observed within the bipolar/rod-shaped microglia. Furthermore, the aligned bipolar/rod-shaped microglia showed no direct contact with other glia cells such as oligodendrocytes and astrocytes after brain injury (Ziebell et al., 2012). The trains of bipolar/rod-shaped microglia aligned end-to-end along the injured axons, which was consistent with other studies (Nissl, 1899; Spielmeyer, 1922; Cho et al., 2006). Nevertheless, how these trains of bipolar/rod-shaped microglia are formed has yet to be elucidated, as does their functional role, particularly their association with neuronal processes.

## Bipolar/Rod-Shaped Microglia in Alzheimer’S Disease

AD is one of the most common neurodegenerative diseases and affects approximately 48 million people worldwide. Aberrant beta amyloid precursor protein (βAPP) metabolism and the subsequent increased deposition of β-amyloid peptides in senile plaques is the major pathological hallmark of AD (Selkoe, 2000). In the early stages of AD pathology, microglia recruited to the senile plaques show strong phagocytic activity and are able to internalize β-amyloid peptides via the class A scavenger receptor (Shaffer et al., 1995; Paresce et al., 1996, 1997). Microglia secrete proteolytic enzymes such as neprilysin, matrix metalloproteinase 9 (MMP-9), plasminogen and insulin-degrading enzyme (IDE), which are capable of degrading β-amyloid peptides (Leissring et al., 2003; Yan et al., 2006). Activated microglia are always detected in the senile plaque core of AD patients (Wegiel and Wisniewski, 1990; Dickson et al., 1993). In a mouse model of AD, the massive infiltration of bone marrow-derived microglia into the senile plaque core significantly disrupted β-amyloid aggregation and reduced the formation of senile plaques in the AD brain (Simard et al., 2006). Genetically modified mice with limited microglia recruitment to the senile plaques during the early onset of AD markedly elevated β-amyloid peptide levels and increased mortality in a transgenic mouse model of AD (El Khoury et al., 2007). These studies further demonstrate the neuroprotective roles of microglia within the early stages of AD pathogenesis. Nevertheless, microglia are known to secrete neurotoxic factors such as IL-1β, NO and TNFα following prolonged exposure to accumulated β-amyloid peptides (Dickson et al., 1993; Barger and Harmon, 1997; Hickman et al., 2008). These activated microglia also exert strong neurotoxicity to hippocampal neurons both *in vitro* (Giulian et al., 1996; Barger and Harmon, 1997) and *in vivo* (Giulian et al., 1995). In another study, the expression of β-amyloid-binding receptors and β-amyloid-degrading enzymes in the microglia of 14-month-old AD mice were markedly reduced as compared to age-matched wild type littermates; meanwhile, expression of the pro-inflammatory cytokines, IL-1β and TNFα, were elevated in microglia (Hickman et al., 2008). This suggests that the phagocytic activity of microglia is significantly impaired upon prolonged exposure to β-amyloid. Studies have also shown that β-amyloid peptides also stimulate astrocytes which greatly reduces the secretion of pro-inflammatory cytokines and NO from β-amyloid-treated microglia (von Bernhardi and Eugenín, 2004). The phagocytic capability of microglia to clear senile plaques was inhibited during co-culture with astrocytes (DeWitt et al., 1998). These findings account for the progressive deposition of β-amyloid peptides even though abundant microglia are present in senile plaques. The accumulation of β-amyloid plaques cause massive neuronal loss, resulting in subsequent cognitive impairments, as demonstrated in AD patients and animal models of AD (West et al., 1994; Casas et al., 2004; Oakley et al., 2006).

Another major pathological hallmark of AD is the deposition of NFTs, which are aggregates of a hyper-phosphorylated form of tau (Ballatore et al., 2007; Ittner and Götz, 2011). Under normal physiological conditions, the expression of tau appeared to be most abundant in axons with some expression in the dendrites (Ittner et al., 2010; Ittner and Götz, 2011). Tau binds to the axons to stabilize the microtubules and promote microtubule assembly (Weingarten et al., 1975; Kadavath et al., 2015), and regulate microtubule-dependent axonal transport via the modulation of motor proteins including dynein and kinesin (Dixit et al., 2008; Scholz and Mandelkow, 2014). Under the pathological condition of AD, however, tau becomes increasingly phosphorylated which causes tau to become detached from the microtubules (Ballatore et al., 2007). The increased level of unbound tau in the cytoplasm increases the likelihood of tau misfolding, resulting in the aggregation and fibril formation of tau (Ross and Poirier, 2004; Kuret et al., 2005a,b). In transgenic mice expressing human mutant tau (P301L), profound microglial activation was observed in the dentate gyrus with extensive degeneration of the axonal terminals. The degenerating axonal terminals, and their synapses, were wrapped by activated microglia. In parallel, a significant reduction of synapsin-I (a synaptic vesicle marker) and postsynaptic density protein 95 (PSD-95, a postsynaptic marker) immunoreactivity indicated a massive loss of synapses during disease progression (de Calignon et al., 2012).

Similar tau pathology was also observed in a transgenic mouse model expressing a different mutant form of human tau (P301S). Tau pathology began at 6-months of age in the P301S tau mutant mice and widespread neuronal loss was detected in these mutants by 8-months of age; microglial activation was detected as early as in the 4-month-old mice. The hippocampal neurons of 4-month-old mutant mice exhibited high levels of immunoreactivity to pro-inflammatory cytokine IL-1β. Administration of the immunosuppressant FK506 to these mutant mice markedly attenuated the inflammatory responses resulting in a significant reduction of activated microglia, tau pathology and neuronal loss (Yoshiyama et al., 2007). Microglia activation has also been commonly observed in transgenic animal models of AD expressing other forms of mutant tau (Ikeda et al., 2005; Zilka et al., 2009; Bhaskar et al., 2010).

Several lines of evidence suggest that chronic inflammation in the brain is likely to exacerbate the formation of NFT and disease-related pathology (Kitazawa et al., 2004; Maccioni et al., 2010). Following exposure to soluble βAPP, the levels of pro-inflammatory cytokine IL-1β was elevated in cultured primary microglia. Co-culturing the primary cortical neurons with βAPP-treated microglia resulted in a substantial increase in tau phosphorylation. However, co-culturing primary neurons with activated microglia pre-treated with an IL-1 receptor (IL-1R)-blocking antibody significantly reduced the phosphorylation of tau in neurons, suggesting that the elevated IL-1β level in activated microglia plays a pivotal role in augmenting tau phosphorylation (Li et al., 2003). In a transgenic mouse model of AD, the *in vivo* administration of LPS induced significant levels of inflammation throughout the cerebral cortex and hippocampus, as well as microglial activation and an increased level of IL-1β, thus resulting in increased tau phosphorylation while the level of β-amyloid remained unchanged (Kitazawa et al., 2005). The activity of Cdk5, a potent mediator of tau phosphorylation (Town et al., 2002), was also significantly elevated following LPS treatment (Kitazawa et al., 2005). In other studies, the selective blockade of IL-1β-mediated signaling pathways using IL-1R blocking antibodies (Kitazawa et al., 2011) or the Cdk5 inhibitor roscovitine (Kitazawa et al., 2005) markedly reduced tau phosphorylation via suppression of the tau kinase p25 activity.

Ablation of Cx3cr1 in mice results in increased microglial activation which exaggerates LPS-induced tau phosphorylation and tau pathology-related behavioral abnormalities including the loss of motor coordination, motor deficits and memory loss (Bhaskar et al., 2010). In contrast, the overexpression of Cx3cr1 in mice suppresses microglial activation and tau phosphorylation. Subsequent neuronal loss in the hippocampus was greatly reduced in mice overexpressing Cx3cr1 (Nash et al., 2013). Pre-treating primary cortical neurons with an IL-1R antagonist could also prevent neurons from the hyper-phosphorylation of tau following exposure to a conditioned medium derived from Cx3cr1-deficient microglia (Bhaskar et al., 2010). Collectively, these studies suggested that microglial activation contributes to the augmentation of tau phosphorylation and its subsequent pathogenesis and that IL-1β is one of the most important pro-inflammatory cytokines responsible for the induction of tau phosphorylation following microglial activation.

In AD patients, infiltrated microglia typically adopt amoeboid morphology (McGeer et al., 1988; Haga et al., 1989; Itagaki et al., 1989); similar findings have been demonstrated in mouse models of AD (Wegiel et al., 2003, 2004; Simard et al., 2006). Amoeboid microglia are mainly localized in the hippocampus and cerebral cortex, where β-amyloid aggregates are formed (Simard et al., 2006), and are responsible for the clearance of β-amyloid aggregates (Shaffer et al., 1995; Paresce et al., 1996). Interestingly, some of the activated microglia adopt an elongated, and highly polarized rod-like morphology, within or in close proximity to senile plaques in patients (Wierzba-Bobrowicz et al., 2002). Trains of bipolar/rod-shaped microglia were predominantly aligned end-to-end in the CA1 and CA2/3 regions of AD hippocampus (Bachstetter et al., 2015). The presence of a characteristic train of bipolar/rod-shaped microglia in an animal model of experimental diffuse traumatic brain injury demonstrated the potential key roles of bipolar/rod-shaped microglia in neuronal survival (Ziebell et al., 2012). Microglial were aligned parallel to damaged neuronal fibers but without physical contact (Bachstetter et al., 2015). In the cerebral cortex of patients with AD, a subset of microglia showed strong immunoreactivity to tau; this subset showed a rod-like morphology with elongated processes (Odawara et al., 1995). The presence of tau-positive bipolar/rod-shaped microglia remained unexplained but may have been due to the internalization of tau-positive degenerated axonal terminals, as significant synaptic loss was observed in the progression of tau pathology (de Calignon et al., 2012). Following the administration of LPS in mice, bipolar/rod-shaped microglia were observed in the cerebral cortex and hippocampus where the levels of phosphorylated tau remained high (Lee et al., 2010). Our early studies also showed that bipolar/rod-shaped microglia expressed a significantly lower level of IL-1β compared with amoeboid microglia (Tam and Ma, 2014). The presence of bipolar/rod-shaped microglia might attenuate the substantial increase in tau phosphorylation induced by microglial activation, and subsequent neuronal loss and functional abnormalities during the progression of AD. Taken together, these findings suggest that bipolar/rod-shaped microglia might be involved in the pathogenesis of AD, or even in the repair process during disease progression (Wierzba-Bobrowicz et al., 2002).

## Bipolar/Rod-Shaped Microglia in Parkinson’S Disease

PD is the second most common neurodegenerative disorder after AD and affects approximately 10 million people worldwide (Dorsey et al., 2007; Delenclos et al., 2016). The cause of PD remains largely unknown since only about 5%–10% of PD patients are related to genetic mutations (Toulouse and Sullivan, 2008), and more than 90% of PD patients remain idiopathic. The pathological features of PD include the widespread loss of dopaminergic neurons in the substantia nigra, resulting in the loss of ascending axonal projections to the striatum. In fact, approximate 50% of dopaminergic neurons are lost and 80% of striatal dopamine is depleted by the time patients are diagnosed with PD (Toulouse and Sullivan, 2008; Long-Smith et al., 2009).

Accumulating evidence suggests that chronic inflammation plays an indispensable role in the degeneration of dopaminergic neurons. Activated microglia accumulate in the substantia nigra of PD patients, where significant neuronal death has been observed (McGeer et al., 1988; Hirsch et al., 1998; Ouchi et al., 2005, 2009; Gerhard et al., 2006). The accumulated microglia are mainly localized in the degenerating neurons. Many pro-inflammatory cytokines, including TNFα, IL-1β, IFNγ and IL-6, have been detected in the brain, as well as in the cerebrospinal fluid and blood plasma, of PD patients (Mogi et al., 1994a,b; Müller et al., 1998; Mount et al., 2007). The combined effects of pro-inflammatory cytokines, including TNFα, IL-1β and IFNγ, have been demonstrated to stimulate the production of NO in a microglia cell line BV-2 (Sheng et al., 2011). NO produced by microglia after treated with LPS and IFNγ induced significant neuronal loss as demonstrated in primary neuron-glia co-culture (Dawson et al., 1994) and in rat hippocampal slice cultures (Papageorgiou et al., 2016). In PD patients, accumulated microglia showed strong inducible NO synthase (iNOS), and cyclooxygenase-2 (Cox-2) immunoreactivity (Hunot et al., 1996; Knott et al., 2000). The elevated level of iNOS and NO production in microglia was associated with the progressive loss of dopaminergic neurons in culture (Le et al., 2001) and in an animal model of PD (Liberatore et al., 1999; Dehmer et al., 2000; Kokovay and Cunningham, 2005; Aquilano et al., 2008). Cox-2 mediates microglial activation and the subsequent secondary neuronal death of dopaminergic neurons since the inhibition of Cox-2 significantly reduced microglial activation and dopaminergic neuronal loss in an animal model of PD (Vijitruth et al., 2006). These studies demonstrated the causal relationship between the prolonged activation of microglia and the subsequent loss of dopaminergic neurons during the pathogenesis of PD.

The major cause for the activation of microglia resulting in prolonged inflammation in PD patients has remained elusive, but evidence suggests that factors released by dying neurons might be responsible (Long-Smith et al., 2009). Several factors released by injured neurons can trigger microglial activation, including α-synuclein aggregates (Zhang et al., 2005; Daniele et al., 2015; Kim et al., 2016), MMP-3 (Kim et al., 2005, 2007) and neuromelanin (Wilms et al., 2003). The presence of such factors in the microglial microenvironment not only induces activation of microglia, but also the secretion of neurotoxic factors which leads to further neuronal cell death during the progression of PD (Kim and Joh, 2006).

In the later stages of PD, bipolar/rod-shaped microglia have been found in close proximity to degenerating dopaminergic neurons in the substantia nigra of patient (McGeer et al., 1988). In a rat model of PD, 3 days after LPS infusion (an animal model to induce neuroinflammation and substantial loss of dopaminergic neurons in the substantia nigra), profound activation of microglia was found in the substantia nigra ipsilateral to the LPS infusion. At this early time point, most microglia adopted bipolar/rod-shaped morphology with enlarged cell bodies and elongated processes. One week after LPS infusion, bipolar/rod-shaped microglia were transformed into amoeboid microglia, and such activation of microglia persisted up to 8 weeks after LPS infusion. The transformation of bipolar/rod-shaped microglia into amoeboid microglia was correlated with significant neuronal loss during the later stages (i.e., 4–6 weeks after LPS infusion; Gao et al., 2002). Amoeboid microglia are known to secrete neurotoxic factors to induce neuronal cell death (Gao et al., 2003; Qin et al., 2004). Interestingly, bipolar/rod-shaped microglia were observed in the activated microglial population (CD11b-positive) 24 h after treatment with aggregated α-synuclein (a pathological hallmark of PD; Zhang et al., 2005). However, whether the existence of bipolar/rod-shaped microglia in PD brains is neuroprotective remains elusive.

## Bipolar/Rod-Shaped Microglia in Huntington’S Disease

HD refers to an inherited genetic disorder characterized by the substantial loss of neurons in medium-sized spiny neurons within the corpus striatum and cerebral cortex (Vonsattel et al., 1985; Vonsattel and DiFiglia, 1998). The classic hallmark of neuropathology in HD is neuronal loss with nuclear and cytoplasmic inclusion of mutant Huntingtin protein and polyglutamine in degenerating neurons (Davies et al., 1997). Several studies suggest that microglia activation shows strong correlation with the pathogenesis of HD (Singhrao et al., 1999; Sapp et al., 2001; Pavese et al., 2006; Björkqvist et al., 2008; Politis et al., 2011; Crotti et al., 2014). Amoeboid microglia were mostly observed throughout the whole corpus striatum as well as the frontal and parietal lobe of the cerebral cortex in HD patients (Sapp et al., 2001; Pavese et al., 2006). The number of microglia was increased within the area where substantial neuronal loss was also detected (Sapp et al., 2001). A recent inflammatory profiling study provided further support for the association between the number of microglia and HD progression. Elevated levels of pro-inflammatory cytokines (IL-1β and TNFα) were detected in the corpus striatum. The level of other inflammatory mediators, such as IL-6, IL-8 and MMP-9, was altered in HD patients with chronic inflammation (Silvestroni et al., 2009). This indicated that the overproduction of pro-inflammatory factors by activated microglia induced neurotoxicity in neurons, thus resulting in neuronal cell death.

In the corpus straitum of HD patients, where the majority of microglia adopted amoeboid morphology, bipolar/rod-shaped microglia have been found aligned along the dendrites of pyramidal neurons in less affected regions of the cerebral cortex as well as in the cerebellum where the dendrites of Purkinje cells and the axonal projections of granule cells were mainly found. The elongated processes of bipolar/rod-shaped microglia were in close contact with the neuronal soma and their adjacent axons (Sapp et al., 2001). Bipolar/rod-shaped microglia showed high immunoreactivity to thymosin β4 and CR3/43, which are both specific for reactive microglia (Graeber et al., 1994).

## Bipolar/Rod-Shaped Microglia in Other Neurodegenerative Diseases

Glaucoma is a neurodegenerative disease characterized by the loss of retinal ganglion cells and subsequent axonal degeneration in the optic nerve (Quigley et al., 1981, 1988, 1989). As with AD and PD, the prolonged activation of microglia contributes, at least in part, to the pathology of glaucoma (Neufeld, 1999; Yuan and Neufeld, 2001; Bosco et al., 2008). In a mouse ocular hypertension (OHT) model of glaucoma, bipolar/rod-shaped microglia were observed in the OHT eyes, but not in the contralateral side which appeared to adopt a ramified morphology. The processes of bipolar/rod-shaped microglia were in close proximity to each other and aligned end-to-end in the retinal nerve fiber layer (NFL). Bipolar/rod-shaped microglia expressed high levels of phagocytic markers (CD68/ED-1 and MHC-II) but relatively low levels of M1 (CD86) and M2 (Ym1) markers (Ziebell et al., 2012; de Hoz et al., 2013). In contrast, low levels of MHC-II immunoreactivity were detected in ramified microglia in the non-injured eyes (de Hoz et al., 2013). This indicates that bipolar/rod-shaped microglia are immunophenotypically distinct from other forms of microglia.

In a rat model of optic nerve transection, bipolar/rod-shaped microglia aligned end-to-end almost exclusively in the ganglion cell layer (GCL) and NFL 7 days after injury. This phenomenon became more pronounced at day 14 and 21 after injury, and the bipolar/rod-shaped microglial alignments disappeared 6 weeks after injury. The bipolar/rod-shaped microglia aligned along the βIII-tubulin-positive neuronal fibers and exhibited strong phagocytic activity to actively internalize degenerating axons (Yuan et al., 2015). Following 10-min focal middle cerebral artery (MCA) ischemia in rats, bipolar/rod-shaped microglial alignments were mainly found in the cerebral cortex around the infarct after 48 and 72 h of reperfusion. However, a more severe injury caused by 120-min focal MCA ischemia induced the microglia to adopt an amoeboid-like phenotype (Zhan et al., 2008). Bipolar/rod-shaped microglia are in a close association with injured neuronal processes, suggesting that they might be involved in the maintenance of neuronal circuitry in the development of neurodegenerative diseases.

## Possible Involvement of Bipolar/Rod-Shaped Microglia in “Synaptic Stripping”

There is a growing body of evidence regarding the bipolar/rod-shaped microglia aligned end-to-end in close proximity along injured axons at an early stage after CNS injury (Zhan et al., 2008; Ziebell et al., 2012; de Hoz et al., 2013; Taylor et al., 2014; Yuan et al., 2015). The trains of bipolar/rod-shaped microglia have prompted researchers to consider their involvement in the reorganization of neuronal circuitry following CNS injury.

The active removal of synaptic terminals by microglia was first observed in the facial nerve injury model by Blinzinger and Kreutzberg (1968), and is now referred to as “synaptic stripping”. Following facial nerve transection in rats, microglia underwent rapid proliferation at the lesion site and migrated to the cell bodies of damaged motor neurons (Graeber et al., 1988) to remove the synaptic boutons from injured neurons (Blinzinger and Kreutzberg, 1968). Synaptic stripping usually takes place within the first few days after injury by interposing the fine microglial processes to the junction between pre-synaptic elements and the post-synaptic cell soma (Moran and Graeber, 2004). Synaptic stripping in the cortical region has also been observed during inflammation induced by heat-killed bacteria in rats. Upon microglial activation, the microglial processes wrap around the cell bodies of cortical neurons, and extend their processes to the axons. Electron microscopy imaging revealed that the synaptic terminals were lost while the microglial processes were in direct contact with the neuronal soma, further suggesting the potential role of microglia in the removal of dysfunctional synapses (Trapp et al., 2007).

Over the last decade, advances in *in vivo* two-photon imaging have enabled the direct visualization of interactions between fluorescently-labeled neurons and microglia in the living brain of genetically modified mice. The processes of ramified microglia made very brief, but direct, contact with the synaptic terminal once per hour. However, the interactions between microglial processes and pre-synaptic boutons were dramatically increased following transient cerebral ischemia. Such a prolonged microglia-synapse interaction usually resulted in the disappearance of pre-synaptic terminals. This suggests that microglia actively detect synaptic conditions, and are involved in synaptic stripping and the subsequent remodeling of neuronal circuitry (Wake et al., 2009).

The specific spatial arrangement of bipolar/rod-shaped microglia after injury indicates their strong association with damaged axons; however, whether this form of microglia is involved in synaptic stripping remains uncertain. Nevertheless, it is noteworthy to mention that microglia morphology varies significantly across different brain regions. In normal adult mouse brain, bipolar/rod-shaped microglia with elongated cell bodies and extremely long primary processes are mainly localized in axon-rich white matter regions including the corpus callosum (i.e., the largest white matter structure in the brain), the molecular layer of the cerebellum (which only consists of axonal projections from Purkinje cells) and the fimbria area in the hippocampus. Bipolar/rod-shaped microglia align in parallel to the adjacent axons (Lawson et al., 1990). The molecular determinants which direct the microglia to adopt a bipolar/rod-shaped phenotype in these brain regions remain poorly understood. However, it is plausible to reason that bipolar/rod-shaped microglia might participate in experience-dependent remodeling and the elimination of synapses, given that they are in such a close spatial association with axonal terminals and the dominance of bipolar/rod-shaped microglia within the axon-rich white matter region (Tremblay et al., 2010). More importantly, the structural removal of impaired synapses from injured axons might be essential to the entire neuronal circuitry in order to make room for the establishment of new connections. This might facilitate the injured nervous system to restore original function after damage (Hanisch and Kettenmann, 2007), but this will require further research.

## Future Perspective: Neuroprotective Roles of Bipolar/Rod-Shaped Microglia

Inflammation-mediated neurodegenerative diseases usually share convergent mechanisms to amplify the inflammatory responses which result in neurotoxicity and subsequent neuronal cell death. Sustained microglia activation is the pathological hallmark of many neurodegenerative diseases which involve the secretion of neurotoxic factors from microglia thus resulting in neuronal loss during the early stages of disease onset (Glass et al., 2010). Interestingly, the presence of bipolar/rod-shaped microglia in the affected region usually takes place at the initial phase of microglial activation before the disease advances from early to later stages. During the end stage of disease, bipolar/rod-shaped microglia can be found in the less affected regions while amoeboid microglia are predominantly seen in the affected regions. This implies that bipolar/rod-shaped microglia might induce neuroprotection and slow down the progression of disease.

Despite the fact that persistent microglial activation is harmful to neurons, as demonstrated in many neurodegenerative diseases, the transient activation of bipolar/rod-shaped microglia might be beneficial in CNS damage to facilitate post-injury neuronal repair mechanisms. Brain injury is known to induce the transient formation of trains of bipolar/rod-shaped microglia at the injury site during the initial phase of injury (Zhan et al., 2008; Ziebell et al., 2012; Taylor et al., 2014). Previous studies by ourselves, and others, have shown that this form of microglia is highly proliferative (Suzumura et al., 1991; Wierzba-Bobrowicz et al., 2002; Tam and Ma, 2014). Inhibition of microglia proliferation at the site of injury showed augmentation of brain damage in the cerebral cortex following ischemic insult in mice (Denes et al., 2007; Lalancette-Hébert et al., 2007). These studies collectively suggest that the initial activation of microglia, and the transient formation of highly proliferative bipolar/rod-shaped microglial alignments, might be essential for expanding the microglial milieu at the site of injury, and exert potential neuroprotection to limit secondary damage to the CNS. Further studies on inhibiting such proliferation and formation of bipolar/rod-shaped microglia alignments in response to diffuse brain injury (Ziebell et al., 2012; Taylor et al., 2014) will be required to elucidate the neuroprotective roles of bipolar/rod-shaped microglia in brain injury.

Bipolar/rod-shaped microglia expressed relatively lower levels of pro-inflammatory cytokines (TNFα and IL1-β) compared to amoeboid microglia (Tam and Ma, 2014). However, upon LPS stimulation, bipolar/rod-shaped microglia were quickly transformed into amoeboid microglia expressing high levels of pro-inflammatory cytokines within a very short period of time (Tam and Ma, 2014; Tam et al., 2016). These “transformed” amoeboid microglia also regained proteolytic properties to degrade laminin (Tam et al., 2016), an extracellular matrix protein which promotes axonal regeneration. Earlier, we stated that bipolar/rod-shaped microglia are usually present during the early phase of neurodegenerative diseases when inflammation is not prominent, while almost exclusively amoeboid microglia have been found in pathological brain at the very late stage of disease onset when neuroinflammation persisted. Further studies should focus on the possibility of bipolar/rod-shaped microglia being transformed into amoeboid microglia during disease progression in animal models. We strongly believe that by transforming the amoeboid microglia into more neuroprotective bipolar/rod-shaped microglia might offer a promising new therapeutic strategy for neurodegenerative diseases and disorders involving chronic neuroinflammation. Our previous study demonstrated that bipolar/rod-shaped microglia expressed high levels of anti-inflammatory cytokines such as IL-10 and TGF-β (Tam and Ma, 2014). IL-10 is known to inhibit the production of pro-inflammatory cytokines (Sawada et al., 1999). Transforming amoeboid microglia back into bipolar/rod-shaped microglia might be a possible strategy to reduce further damage to CNS neurons by minimizing the production of neurotoxic cytokines.

Accumulating evidence also suggests that the removal of dysfunctional synaptic terminals by microglia is of great importance for subsequent neuronal repair. Followed by facial nerve injury, the loss of synapses appeared to be one of the earliest responses brought about by microglial activation (Blinzinger and Kreutzberg, 1968; Moran and Graeber, 2004). This may be considered as a neuroprotective response since the removal of dysfunctional synapses prevented injured neurons from excitotoxicity leading to better neuronal survival (Wake et al., 2009; Kato et al., 2016). The preserved motor neurons then reactivate their intrinsic growth program to regrow injured axons, and to reconnect with the original target muscle for functional synapse formation (Wake et al., 2009; Perry and O’Connor, 2010). Recent investigations on microglia-neuron interactions using *in vivo* two-photon imaging and patch clamp methods further support the neuroprotective roles of microglia. Microglia actively migrate towards and enwrap the swollen axons induced by neuronal hyperactivity. Pharmacological blockade of microglial migration prevented microglia from physically contacting the swollen axons. The neurons eventually underwent apoptotic cell death due to excitotoxicity (Kato et al., 2016). After optic nerve transection, bipolar/rod-shaped microglia internalized the injured optic nerve fibers and the degenerating retinal ganglion cells (Yuan et al., 2015). The close association between bipolar/rod-shaped microglia and injured axonal fibers might be linked to the reorganization of neuronal circuitry, and subsequent neuronal regenerative processes. Chronic neuroinflammation usually results in significant neuronal loss. Therefore, by switching microglial morphology from amoeboid form to bipolar/rod-shaped microglia might help to actively remove the impaired synaptic clefts and injured axons, thus creating more favorable conditions for axonal regrowth and the re-establishment of new synaptic connections.

**Figure 2:**
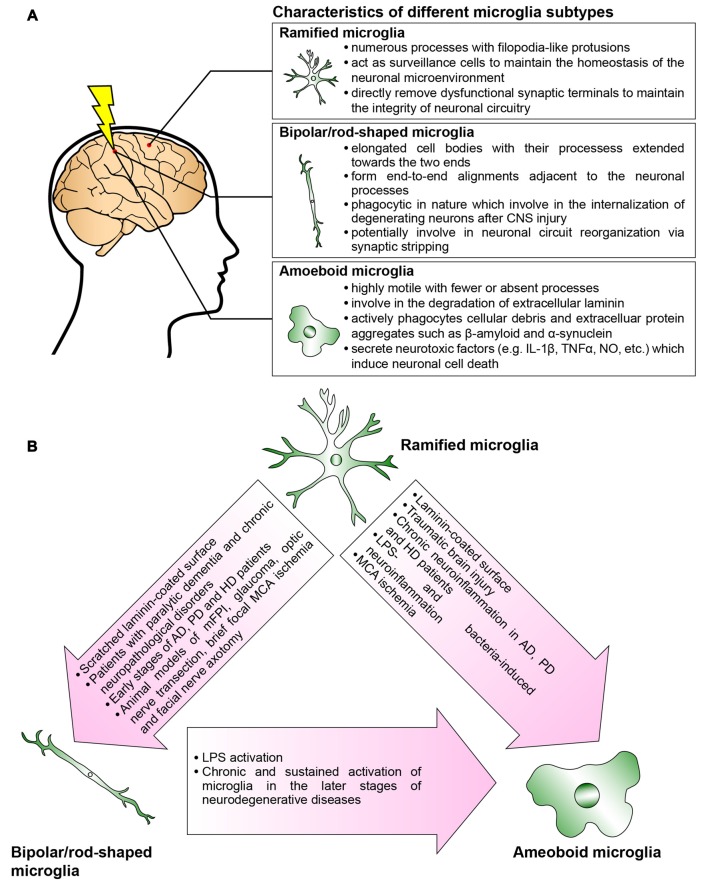
**Microglia are phenotypically dynamic. (A)** The functional roles of microglia in normal or pathological central nervous system (CNS) are tightly associated with their morphological changes. Ramified microglia actively sense the subtle changes in CNS microenvironment. Bipolar/rod-shaped microglia form end-to-end alignments in close proximity to damaged neuronal fibers, suggesting that their functions are related to synaptic reorganization. Amoeboid microglia are extremely motile which actively migrate towards the injury site. They are highly phagocytic to internalize cellular debris and secrete neurotoxic factors which induce neuronal cell death. However, there are microglia subtypes that have yet not been fully characterized (i.e., hyper-ramified, bushy-like and spider-like microglia; Karperien et al., 2013; Ziebell et al., 2015). **(B)** In normal healthy CNS, most of the microglia are displayed as ramified microglia. In response to the change in the microenvironment, the ramified microglia undergo rapid transformation into bipolar/rod-shaped microglia or amoeboid microglia depending on the types of stimulus. Bipolar/rod-shaped microglia can quickly transform into amoeboid microglia in response to lipopolysaccharide (LPS) activation. The amoeboid microglia transformed from bipolar/rod-shaped microglia secrete pro-inflammatory cytokines and degrade extracellular laminin.

Collectively, an in-depth understanding of the spatial and temporal activation of bipolar/rod-shaped microglia, and their association with degenerating axons, would render them excellent candidates with which to devise new strategies for treating neurodegenerative diseases. The recent establishment of a cost-effective and highly reproducible *in vitro* cell culture system to enrich bipolar/rod-shaped microglia allows more comprehensive studies on their gene and secretory protein expression profiles (Tam and Ma, 2014; Tam et al., 2016). It would be of great interest to explore if bipolar/rod-shaped microglia could also secrete neurotrophic factors such as brain-derived neurotrophic factor (BDNF), nerve growth factor (NGF) and neurotrophin-3 (NT-3; Nakajima et al., 2001), which contribute to the promotion of neuronal survival following injury and neurodegeneration (Hanisch and Kettenmann, 2007). Further research is now required to elucidate the mechanisms underlying the regulation of transformation from bipolar/rod-shaped microglia into amoeboid microglia and how this morphological change can be reversed to the bipolar/rod-shaped or ramified microglia (Figure 2). More importantly, the development of new therapeutic interventions by switching the microglial phenotype from amoeboid to bipolar/rod-shaped microglia might shed new light on pathogenesis and identify targets for treating neurodegenerative diseases.

## Author Contributions

NPBA and CHEM discussed and formed the review focus. NPBA conducted the literature review and wrote the first draft of the manuscript under the supervision of CHEM. CHEM evaluated and revised the manuscript for final submission. All authors have made substantial and intellectual contributions to the current work and approved the final version for submission.

## Conflict of Interest Statement

The authors declare that the research was conducted in the absence of any commercial or financial relationships that could be construed as a potential conflict of interest.
